# Adjudication of Hospitalizations and Deaths in the IRONMAN Trial of Intravenous Iron for Heart Failure

**DOI:** 10.1016/j.jacc.2024.08.052

**Published:** 2024-10-29

**Authors:** John G.F. Cleland, Pierpaolo Pellicori, Fraser J. Graham, Rebecca Lane, Mark C. Petrie, Fozia Ahmed, Iain B. Squire, Andrew Ludman, Alan Japp, Abdallah Al-Mohammad, Andrew L. Clark, Ben Szwejkowski, Chris Critoph, Victor Chong, Rebekah Schiff, Thuraia Nageh, Jason Glover, John J.V. McMurray, Elizabeth A. Thomson, Michele Robertson, Ian Ford, Philip A. Kalra, Paul R. Kalra, IRONMAN Study Group

**Affiliations:** a School of Cardiovascular and Metabolic Health, University of Glasgow, Glasgow, United Kingdom; b Royal Brompton and Harefield Hospitals, London, United Kingdom; c Department of Cardiology, Manchester University NHS Foundation Trust, Manchester, United Kingdom; d Department of Cardiovascular Sciences, University of Leicester, Leicester, United Kingdom; e Royal Devon University Healthcare NHS Foundation Trust, Exeter, United Kingdom; f Royal Infirmary of Edinburgh, Edinburgh, United Kingdom; g Sheffield Teaching Hospitals NHS Foundation Trust, Sheffield, United Kingdom; h University of Sheffield, Sheffield, United Kingdom; i Hull York Medical School, University of Hull, Hull, United Kingdom; j Ninewells Hospital and Medical School, Dundee, United Kingdom; k University Hospitals Dorset NHS Foundation Trust, Bournemouth, United Kingdom; l NHS Ayrshire & Arran, Ayr, United Kingdom; m Guy’s and St Thomas’ NHS Foundation Trust, London, United Kingdom; n Southend University Hospital, Southend, United Kingdom; o Hampshire Hospitals NHS Foundation Trust, Basingstoke, United Kingdom; p Robertson Centre for Biostatistics, University of Glasgow, Glasgow, United Kingdom; q Salford Royal Hospital, Northern Care Alliance NHS Foundation Trust, Salford, United Kingdom; r University of Manchester, Manchester, United Kingdom; s Department of Cardiology, Portsmouth Hospitals University NHS Trust, Portsmouth, United Kingdom; t Faculty of Science and Health, University of Portsmouth, Portsmouth, United Kingdom; u College of Medical, Veterinary and Life Sciences, University of Glasgow, Glasgow, United Kingdom

**Keywords:** heart failure, hospitalizations, intravenous iron, iron deficiency, randomized trial

## Abstract

**Background:**

Patients with heart failure and iron deficiency have diverse causes for hospitalization and death that might be affected by iron repletion.

**Objectives:**

The purpose of this study was to explore causes of hospitalizations and deaths in a randomized trial (IRONMAN) of heart failure comparing intravenous ferric derisomaltose (FDI) (n = 568) and usual care (n = 569).

**Methods:**

Patients with heart failure, left ventricular ejection fraction ≤45%, and either transferrin saturation <20% or serum ferritin <100 μg/L were enrolled. Median follow-up was 2.7 years (Q1-Q3: 1.8-3.6 years). A committee adjudicated the main and contributory causes of unplanned hospitalizations and deaths. RRs (rate ratios) for selected recurrent events with 95% CIs are also reported.

**Results:**

Compared with usual care, patients randomized to FDI had fewer unplanned hospitalizations (RR: 0.83; 95% CI: 0.71-0.97; *P* = 0.02), with similar reductions in cardiovascular (RR: 0.83; 95% CI: 0.69-1.01) and noncardiovascular (RR: 0.83; 95% CI: 0.67-1.03) hospitalizations, as well as hospitalizations for heart failure (RR: 0.78; 95% CI: 0.60-1.00), respiratory disease (RR: 0.70; 95% CI: 0.53-0.97), or infection (RR: 0.82; 95% CI: 0.66-1.03). Heart failure was the main cause for 26% of hospitalizations and contributed to or complicated a further 12%. Infection caused or contributed to 38% of all hospitalizations, including 27% of heart failure hospitalizations. Patterns of cardiovascular and all-cause mortality were similar for patients assigned to FDI or usual care.

**Conclusions:**

In IRONMAN, FDI exerted similar reductions in cardiovascular and noncardiovascular hospitalizations, suggesting that correcting iron deficiency might increase resistance or resilience to a broad range of problems that cause hospitalizations in patients with heart failure. (Intravenous Iron Treatment in Patients With Heart Failure and Iron Deficiency; NCT02642562)

For patients with heart failure, reduced left ventricular ejection fraction (HFrEF), and iron deficiency, intravenous administration of iron increases hemoglobin, improves symptoms, and reduces hospitalizations for heart failure,^1^, ^2^, ^3^ though evidence for a reduction in cardiovascular (CV) or all-cause mortality remains elusive.^1^

But what constitutes a hospitalization for heart failure?^4^, ^5^, ^6^, ^7^, ^8^, ^9^ Most people would agree that an admission for worsening breathlessness and peripheral edema that responds to intensification of diuretic therapy constitutes a hospitalization for heart failure; but what if worsening heart failure is clearly precipitated by atrial fibrillation, myocardial infarction, worsening renal function, or infection? In clinical practice, several problems often conspire to cause a hospitalization or death,^6^ but clinical endpoint adjudication committees (CEACs) conventionally attribute hospitalizations or deaths to a single cause.^10^ Recognizing that diverse problems often conspire to cause hospitalization or death better reflects clinicians’ experience and helps identify unmet needs and alternative therapeutic targets that should be investigated in future trials.

The IRONMAN (Intravenous Iron Treatment in Patients With Heart Failure and Iron Deficiency; NCT02642562) trial investigated the effects of intravenous ferric derisomaltose (FDI) compared with usual care in patients with HFrEF and evidence of iron deficiency.^11^^,^^12^ The primary endpoint, a composite of recurrent hospital hospitalizations for heart failure and CV death, was of borderline statistical significance. A first-event analysis of all-cause hospitalizations was reported in the primary report and was not statistically significant (HR: 0.91; 95% CI: 0.79-1.05; *P* = 0.21).^11^ We now present an analysis of the main and contributory causes of hospitalizations, using a recurrent-events analysis, and deaths to provide further insights into the pattern of clinical effects of intravenous iron in patients with HFrEF.

## Methods

The IRONMAN trial was an investigator-initiated, randomized, open-label, blinded-endpoint trial conducted in the United Kingdom comparing intravenous FDI and usual care (Central Illustration). The trial was funded by the British Heart Foundation (grant award CS/15/1/31175). Pharmacosmos supplied FDI and gave additional financial support. The trial protocol and amendments were approved by a national ethics committee in the United Kingdom (Leicester South Research Ethics Committee, Integrated Research Application System no. 191168), the Medicines and Healthcare Products Regulatory Agency, and the UK Health Research Authority.^11^ The trial was registered at clinicaltrials.gov (NCT02642562). The design and main results have been published previously.^11^^,^^13^

**Central Illustration:**
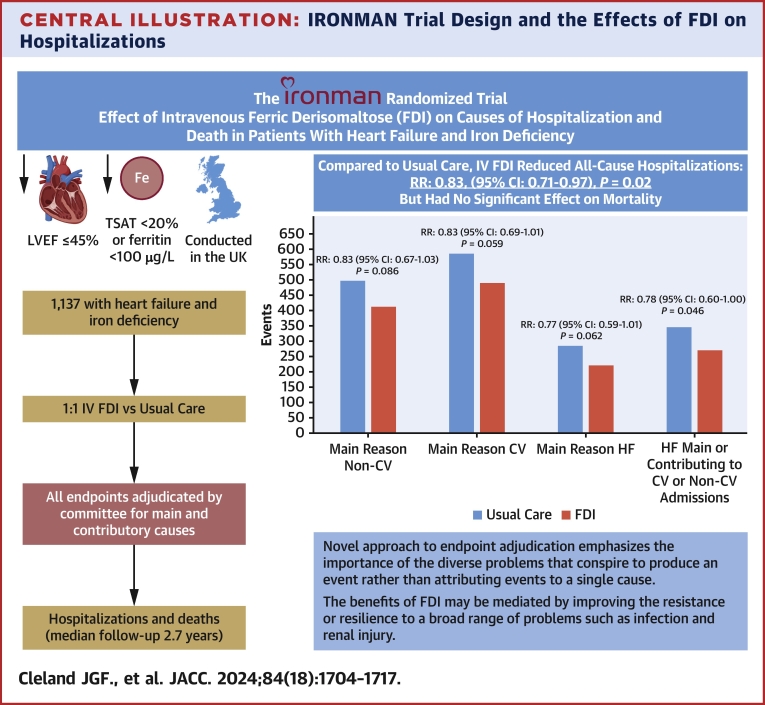
IRONMAN Trial Design and the Effects of FDI on Hospitalizations Design of the IRONMAN trial and effect of FDI on all-cause and cause-specific hospitalizations (recurrent events analysis) for patients with chronic heart failure with a reduced left ventricular ejection fraction (LVEF) and markers of iron deficiency. CV = cardiovascular; IV = intravenous; RR = rate ratio; TSAT = transferrin saturation.

Adults with new or established symptomatic heart failure and LVEF ≤45% were invited to participate. After written informed consent, patients were screened for inclusion and exclusion criteria. Hemoglobin had to be ≥9 g/dL, but <13 g/dL for women and <14 g/dL for men. Serum ferritin had to be <100 μg/L or transferrin saturation (TSAT) <20%; those with a serum ferritin >400 μg/L were excluded. Patients were required to have either a current or recent (<6 mo) heart failure hospitalization or raised plasma concentrations of natriuretic peptides.^13^

Eligible patients were randomly assigned (1:1) to receive FDI or usual care. Up to 20 mg/kg of iron, to a maximum of 2,000 mg, could be administered as a single infusion, depending on hemoglobin concentration and patient’s weight. The protocol requested that participants be reviewed 4 weeks after randomization and every 4 months thereafter until trial completion. For those assigned to FDI, investigators were asked to give further doses of FDI at trial visits if ferritin was <100 μg/L or, if ferritin was ≤400 μg/L, TSAT was <25%.

The primary endpoint was hospitalization for heart failure and CV death analyzed using a recurrent events analysis. Analysis of cause-specific hospitalizations or deaths was not prespecified and the trial was not powered to show such differences. This report should be considered mainly descriptive rather than hypothesis testing.

A CEAC, blind to treatment allocation, adjudicated all unplanned hospitalizations and all deaths. The CEAC comprised 3 cardiologists, F.J.G., P.P., and J.G.F.C., who reviewed case report forms, discharge letters, and death certificates. Initially, all events were reviewed by all 3 members to gain experience and ensure consistency. Thereafter, events were evaluated by a single CEAC member, who could request review by the whole committee in cases of uncertainty. Planned hospitalizations from a waiting list that did not result in serious disability or death (eg, uncomplicated cataract surgery) were not included in this analysis. To avoid including hospitalizations that were precautionary rather than for a major event, hospitalizations were required to be for >24 hours or to be fatal to be considered as endpoints. Record linkage to national databases of hospital discharges and deaths was done to ensure identification of relevant events that investigators might have missed.

Hospitalizations were classified as CV or non-CV. The main cause of hospitalization was then classified as presented in Supplemental Table 1 and contributory CV and non-CV causes recorded. A single event could have many contributory causes. For example, an admission for worsening congestion due to recurrent atrial fibrillation and anemia with evidence of gastrointestinal bleeding while on an oral anticoagulant would be adjudicated as hospitalization mainly for heart failure with 3 contributory CV causes, that is, atrial fibrillation, bleeding, and CV therapy. Hospitalization was considered to be due to heart failure if worsening symptoms or signs of congestion required intensified diuretic therapy. When worsening heart failure was the main cause of hospitalization or made an important contribution to hospitalization for another CV cause, such as atrial fibrillation, it was considered to be a primary endpoint. However, when heart failure was contributory to non-CV hospitalization, it was not. Heart failure occurring late after hospital hospitalization was also recorded but did not count toward the primary endpoint. For the primary endpoint, hospitalizations for heart failure during which the patient died from CV causes were counted as a single event, but hospitalization and deaths are counted separately in the present report.

Hospitalizations due to complications of CV therapy, including bleeding or acute kidney injury (AKI), were considered to be CV. Bleeding was considered to be CV because the CV system is designed to contain blood and bleeding is usually caused or exacerbated by antithrombotic therapies in patients with heart failure. Gastrointestinal bleeding was included in this definition. Bleeding due to minor trauma also was considered to be CV, but when due to major trauma was not. AKI was considered to be a cardiorenal event because worsening cardiac function and treatment with diuretics and renin-angiotensin-aldosterone system inhibitors can all cause reversible deterioration in renal function. However, hospitalizations for persistent severe renal dysfunction were considered to be non-CV. Arrhythmias as the main cause for hospitalization were classified as either ventricular or supraventricular, but when contributing to another cause of hospitalization were simply classified as an arrhythmia, without distinguishing between atrial and ventricular. Admissions due to Covid-19 were classified as non-CV due to respiratory infection, but they could have heart failure or other CV complications as contributory reasons.

Deaths were adjudicated similarly but including a category for sudden death (Supplemental Table 1). Deaths preceded by recurrent hospitalizations for heart failure or severe and persistent symptoms were classified as due to heart failure even if the final event was sudden. Deaths could be recorded as sudden in the setting of worsening heart failure where the patient was not yet considered as end-stage. Deaths were classified also according to whether they occurred in hospital, at home, or elsewhere.^14^^,^^15^ Local investigators, rather than the CEAC, reported whether they considered the death to be unexpected.

### Statistical analysis

Categoric data are shown as percentages and continuous data as median (Q1-Q3). Cause-specific hospitalizations and deaths are reported as both number of events and patients with an event; statistical testing was done only selectively for more frequent events. Recurrent event rates for all-cause, CV, and non-CV hospitalizations between treatment arms were compared by means of semiparametric regression of mean functions for the recurrent events, as described by Lin et al,^16^ with the treatment effect expressed as an RR (rate ratio) with 95% CI.^17^ Graphic presentations of the estimated mean numbers of recurrent events over time are plotted based on the method of Ghosh and Lin.^17^ Time-to-first event outcomes were analyzed in a similar manner with the use of Cox proportional hazard models and treatment effects were estimated as HRs with 95% CIs. All analyses were adjusted for the randomization stratification variable, which had 3 categories (in hospital, recent discharge from hospital, and outpatient with high plasma concentrations of natriuretic peptides). All cited *P* values are 2-sided. Data were analyzed with the use of SAS version 9.4, R version 4.3.2, and Minitab version 21.4.2.

## Results

Over a median follow-up of 2.7 years (Q1-Q3: 1.8-3.6 years), of 1,137 patients enrolled, 721 (63%) had at least 1 adjudicated hospitalization and 377 (33%) died.

### Hospitalizations

Table 1 presents the number of hospitalizations (recurrent events) classified by main or contributory causes and the number of patients who experienced such events. Of the 569 patients assigned to usual care, 198 (35%) had no adjudicated hospitalization for the duration of the trial, of whom 27 died during follow-up, 118 (21%) had only 1 hospitalization, 99 (17%) had 2 hospitalizations, and 153 (27%) had 3 or more hospitalizations. For the 568 patients assigned to FDI, 219 (38%) had no adjudicated hospitalization, of whom 21 died during follow-up, 139 (25%) had only 1 hospitalization, 89 (16%) had 2 hospitalizations, and 122 (21%) had 3 or more hospitalizations.

**Table 1:** Causes of Hospitalizations

	Usual Care (n = 568)				Ferric Derisomaltose (n = 569)			
Deaths after randomization without preceding hospitalization	27 Deaths				21 Deaths			

All Hospitalizations	Main Cause		Main or Contributory		Main Cause		Main or Contributory	
	Events	Patients	Events	Patients	Events	Patients	Events	Patients
All causes	1,086	370 (65)	a	a	904	351 (62)	a	a
CV	589 (54)	273 (48)	a	a	491 (54)	254 (45)	a	a
Heart failure	286	152	345	179	222	130	269	152
VT/VF	47	29	a	a	26	22	a	a
AF/SVT	15	14	68^b^	53^b^	20	19	57^b^	43^b^
ACS/MI	38	25	46	33	23	18	30	23
Stroke	10	10	12	12	24	21	29	24
AKI	28	24	193	144	21	19	165	114
Hemorrhage	34	26	68	53	48	39	82	62
Complications of CV therapy	42	37	205	147	36	35	202	142
PTE	3	3	3	3	2	2	2	2
Other	86	69	a	a	69	63	a	a
Non-CV	497 (46)	249 (44)	a	a	413 (46)	214 (38)	a	a
Cancer	18	14	37	21	15	14	47	30
Respiratory	45	31	196	124	29	21	137	93
Infection	213	140	420	221	175	111	345	189
Respiratory and infection^c^	a	a	174	115	NA	NA	130	88
Renal	17	16	88	67	21	19	78	61
Trauma and non-CV procedures	63	54	95	73	48	42	92	76
Other	141	96	a	a	125	94	a	a

Values are n or n (%).

ACS = acute coronary syndrome; AF = atrial fibrillation; AKI = acute kidney injury (see text for explanation of cardiorenal); CV = cardiovascular; MI = myocardial infarction; PTE = pulmonary thromboembolism; SVT = supraventricular tachycardia; VF = ventricular fibrillation; VT = ventricular tachycardia.

a Not appropriate or not calculated.

b Arrhythmias complicating other hospitalizations were not further adjudicated as SVT or VT but were considered to be predominantly SVT.

c Respiratory and infection includes patients with a respiratory admission with infection as contributory or admission for infection with respiratory disease as contributory. This includes patients in the 2 previous rows who had both as either main or contributory causes.

**Figure 1:**
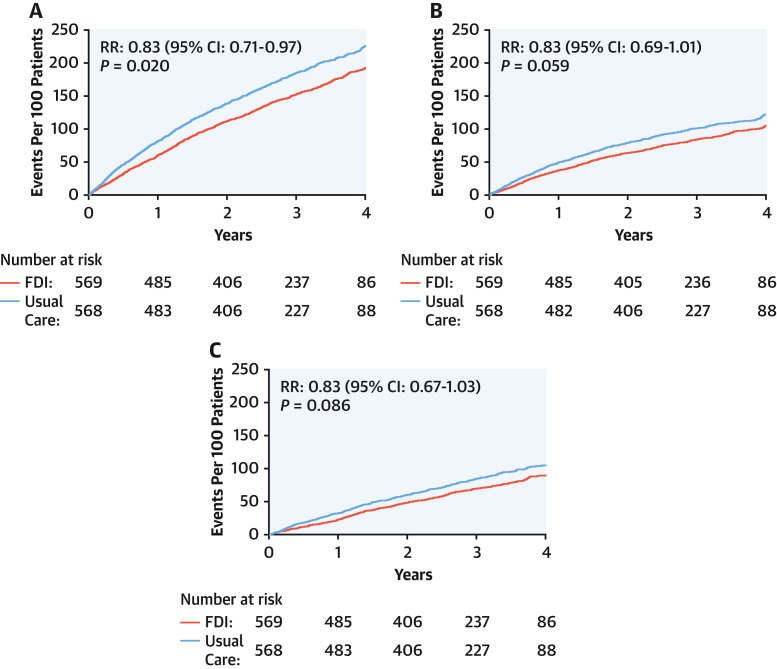
Cumulative Incidence Curves for All-Cause, Cardiovascular, and Noncardiovascular Hospitalizations (Recurrent Events) Graphic presentations of cumulative events for patients assigned to usual care or ferric derisomaltose (FDI), including (A) all-cause hospitalizations and hospitalizations where the main cause was (B) cardiovascular or (C) noncardiovascular based on the method of Ghosh and Lin,^17^ adjusting for the competing risk of death. Reductions in event rates were similar for each type of hospitalization.

**Figure 2:**
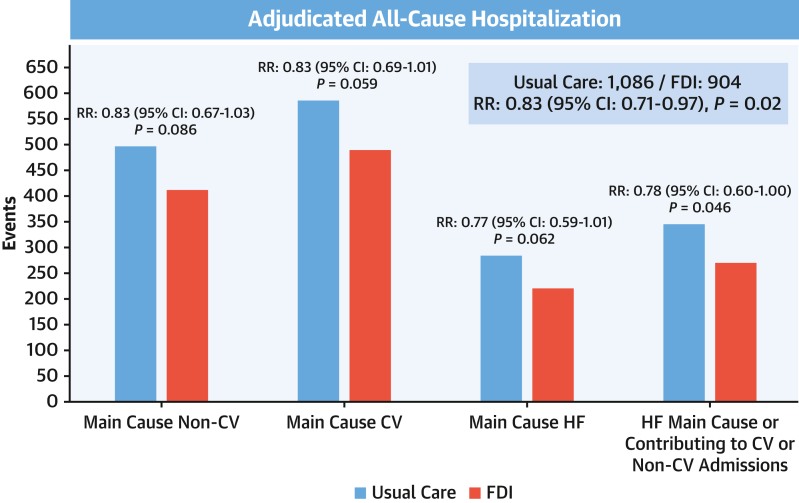
Causes of Hospitalizations Numbers of hospitalizations for noncardiovascular (non-CV) and cardiovascular (CV) causes and for heart failure (HF) as the main cause or as a main cause or contributory to another CV or non-CV cause according to randomized group, ie, usual care or ferric derisomaltose (FDI). Recurrent event rates between treatment arms were compared according to the method of Lin et al.^16^

Compared with usual care, patients randomized to FDI had fewer unplanned all-cause hospitalizations (904 vs 1,086; RR: 0.83; 95% CI: 0.71-0.97; *P* = 0.02), with similar reductions in CV (RR: 0.83; 95% CI: 0.69-1.01) and non-CV (RR: 0.83; 95% CI: 0.67-1.03) hospitalizations (Figure 1, Table 1, Central Illustration). This was also true for heart failure as the main cause of hospitalization (RR: 0.77; 95% CI: 0.59-1.01), when it also contributed to another CV cause (RR: 0.80; 95% CI: 0.62-1.03), and when it contributed to hospitalizations for any other cause (RR: 0.78; 95% CI: 0.60-1.00) (Figure 2). For hospitalizations ascribed mainly to heart failure, AKI, infection, respiratory disease, and arrhythmia were often contributory causes (Table 2).

**Table 2:** Main and Contributory Causes of Hospitalizations Associated With Worsening Heart Failure

	Usual Care			Ferric Derisomaltose		
	HF Main Cause	HF Contributory	HF late Complication^a^	HF Main Cause	HF Contributory	HF late Complication^a^
Main cause	CV: 286	CV: 34 Non-CV: 31	CV: 29^b^ Non-CV: 40	CV: 222	CV: 35 Non-CV: 17	CV: 32^b^ Non-CV: 34
**Cause for Admission**	**Contributory Cause**	**Main Cause When Not HF**	**Main Cause**	**Contributory Cause**	**Main Cause When Not HF**	**Main Cause**
CV						
HF	NA	NA	60^c^	NA	NA	63^c^
VT/VF	d	9	2	d	6	3
AF/SVT	29	3	3	16	3	3
ACS/MI	3	2	6	1	3	4
Stroke	1	0	0	0	1	1
AKI	89	6	1	66	7	4
Hemorrhage	12	1	4	10	3	7
Complications of CV therapy	23	1	5	25	3	3
PTE	0	1	1	0	0	0
Other CV	e	6	7	e	4	7
Non-CV						
Cancer	2	1	0	3	0	1
Respiratory	40	3	3	32	2	3
Infection	78	19	25	60	8	19
Renal	5	0	0	0	1	1
Trauma	0	2	4	5	1	6
Other non-CV	e	6	8	e	5	4

Values are n.

NA = not applicable; other abbreviations as in Table 1.

a Heart failure occurring as a late complication during an admission, including in-hospital worsening of heart failure admissions.

b Where heart failure was considered the main or contributory cause for admission, late exacerbations are not included in this figure, to avoid double-counting.

c Exacerbation of heart failure as a late complication of hospitalizations with heart failure as a main or contributory cause.

d Arrhythmias complicating other hospitalizations were not further adjudicated as SVT or VT but were considered to be predominantly SVT.

e Not included in adjudication algorithm.

**Figure 3:**
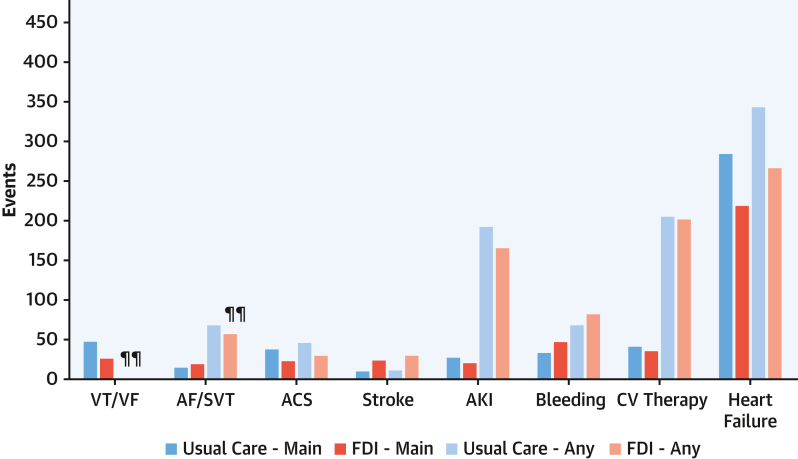
Main or Contributory Causes for Cardiovascular Hospitalizations Number of cardiovascular hospitalizations as a main or contributory cause according to randomized group, ie, usual care or ferric derisomaltose (FDI). ^¶¶^The algorithm did not classify the type of arrhythmia when contributory, but most arrhythmias as a contributory cause were considered to be AF/SVT. Any = main or contributory cause for admission. ACS = acute coronary syndrome; AF/SVT = atrial fibrillation or supraventricular tachycardia; AKI = acute kidney injury; VT/VF = ventricular tachycardia or ventricular fibrillation or cardiac arrest. The *y*-axis scale is the same as in Figures 2 and 3 to allow comparisons.

Apart from heart failure, the most common main and contributory CV causes for hospitalizations were complications of CV therapy, arrhythmias (including cardiac arrest), and bleeding (Figure 3). AKI was rarely considered to be the main cause of hospitalization but was a common contributory cause. Those randomized to FDI tended to have fewer hospitalizations for arrhythmias, acute coronary syndromes, or AKI, but more hospitalizations for bleeding. Hospitalizations for stroke were not common, but they occurred more often among patients assigned to FDI.

**Figure 4:**
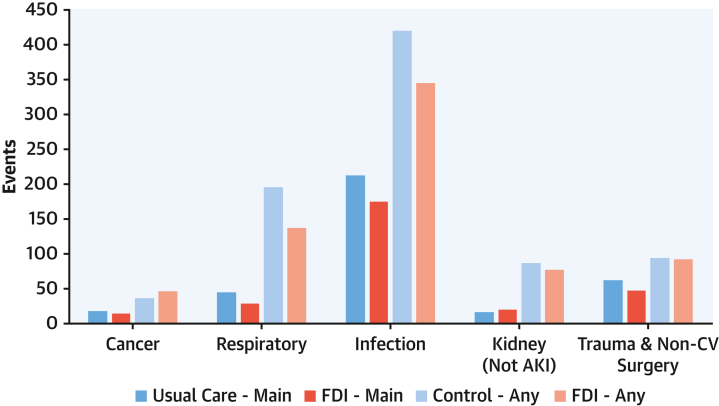
Main or Contributory Causes for Noncardiovascular Hospitalizations Legend: Numbers of noncardiovascular hospitalizations as a main or contributory cause according to randomized group, ie, usual care or ferric derisomaltose (FDI). Any = main or contributory cause for admission. The *y*-axis scale is the same as in Figures 2 and 3 to allow comparisons.

The main non-CV causes for hospitalizations were infections or trauma and non-CV surgery, often for fractures due to falls (Figure 4). When both main and contributory non-CV causes for hospitalization were considered, the picture was dominated by infections and respiratory hospitalizations. Those randomized to FDI had fewer hospitalizations for respiratory disease (RR: 0.70; 95% CI: 0.53-0.97) or infection (RR: 0.82; 95% CI: 0.66-1.03).

### Deaths

**Figure 5:**
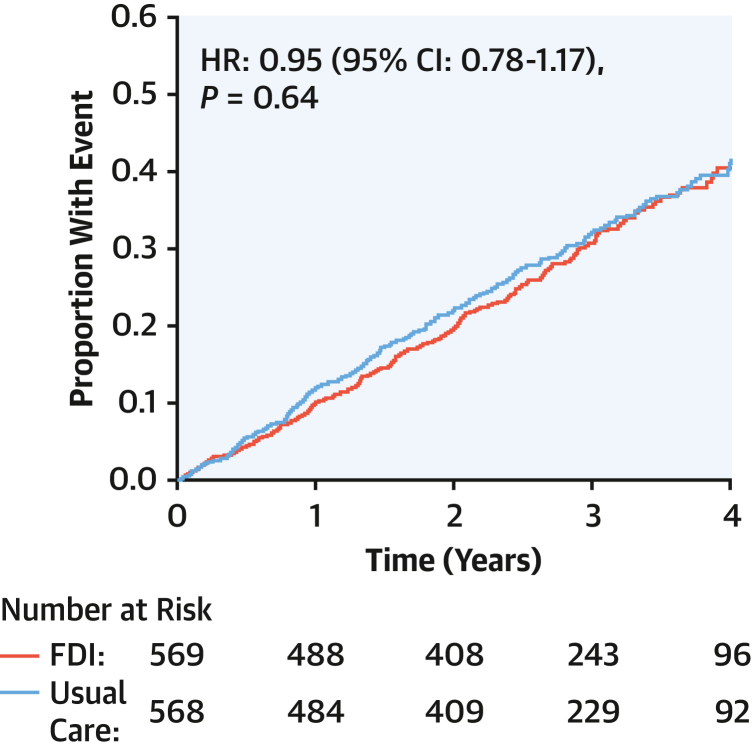
All-Cause Mortality All-cause mortality for patients assigned to usual care or ferric derisomaltose (FDI). HRs (with 95% CIs) were adjusted for the baseline stratification variable of recruitment context (current or recent hospital hospitalization for heart failure or as an outpatient with increased plasma concentrations of natriuretic peptides).

The median age at death was 76 years (Q1-Q3: 69-82 years). Similar numbers of patients died among those assigned to FDI and those to usual care (Figure 5), although there were numerically fewer CV deaths for those randomized to FDI.

The most common causes for CV death were heart failure and sudden death (Table 3). Heart failure was a main or contributory cause for most CV deaths and for more than one-half of all deaths. AKI, respiratory disease, and infection frequently contributed to heart failure deaths. The main causes for non-CV deaths in both randomized groups were infections and cancer.

**Table 3:** Main and Contributory Causes of Death

Deaths	Usual Care				Ferric Derisomaltose			
All-cause	192				184			

	**Main Cause**	**Main or Contributory**	**Contributory to HF Deaths**	**Contributory to Sudden Death**	**Main Cause**	**Main or Contributory**	**Contributory to HF Deaths**	**Contributory to Sudden Death**
CV	138 (72)				119 (65)			
HF	82	110	NA	10	71	107	NA	12
Sudden cardiac	33	35	1	NA	32	40	6	NA
ACS/MI	1	4	2	1	1	4	2	1
Stroke	2	5	2	0	7	7	0	0
AKI	7	53	37	0	1	42	25	0
Hemorrhage	7	10	0	0	2	8	0	0
Complications of CV therapy	0	8	1	0	2	12	1	0
PTE	0	0	0	0	1	1	0	1
Other CV	6	a	a	a	2	a	a	a
Non-CV	55 (28)				65 (35)			
Cancer	16	17	0	0	16	24	2	1
Respiratory	5	49	19	2	5	39	7	2
End-stage kidney disease	0	4	0	0	1	6	0	0
Trauma/non-CV procedures	2	6	2	0	0	5	3	0
Infection	28	71	26	1	34	63	13	3
Other non-CV	4	a	a	a	9	a	a	a

Values are n (%) or n.

Abbreviations as in Tables 1 and 2.

a Not included in adjudication algorithm.

### Composite outcomes of recurrent hospitalizations and CV death

There were 336 primary endpoints in those assigned to FDI and 411 in those assigned to usual care (RR: 0.82; 95% CI: 0.66-1.02; *P* = 0.070). If the primary endpoint had included non-CV hospitalizations with heart failure as a contributory cause, there would have been, respectively, 353 and 440 events (RR: 0.80; 95% CI: 0.65-0.99; *P* = 0.044).

### Mode of death

Of 377 deaths, only 116 were considered unexpected by local investigators, of which the CEAC considered 46 (41%) were sudden (Table 4). Of 65 deaths that the CEAC considered sudden, 45 occurred at home and 11 within 24 hours after hospitalization. Mode of death was similar for patients assigned to usual care and to FDI.

**Table 4:** Place and Mode of Death by Main Cause of Death

Deaths	Control						Ferric Derisomaltose					
	Main Reason for Death	Unexpected^a^	At Home	In Hospital <24 h	In Hospital >24 h	Other	Main Reason for Death	Unexpected^a^	At Home	In Hospital <24 h	In Hospital >24 h	Other
All cause	193	68	49	20	101	23	184	48	58	14	89	23
Cardiovascular	138	53	41	16	63	18	119	40	47	11	50	12
Heart failure	82	23	15	7	47	13	71	13	27	5	34	5
Sudden cardiac	33	24	23	6	1	4	32	22	19	5	3	5
ACS/MI	1	1	1	0	0	0	1	1	1	0	0	0
Stroke	2	1	1	0	1	0	7	1	0	0	7	0
AKI	7	1	0	3	4	0	1	0	0	0	0	1
Hemorrhage	7	2	0	0	6	1	2	0	0	0	2	0
Iatrogenic	0	0	0	0	0	0	2	1	0	0	2	0
PTE	0	0	0	0	0	0	1	0	0	0	1	0
Other CV	6	1	0	1	4	1	2	2	0	1	1	0
Non-CV	55	15	8	4	38	5	65	8	11	3	39	12
Cancer	16	4	7	1	6	2	16	1	6	0	3	7
Respiratory	5	3	0	1	4	0	5	1	0	1	4	0
Renal	0	0	0	0	0	0	1	0	0	1	0	0
Trauma/non-CV procedures	2	1	1	0	1	0	0	0	0	0	0	0
Infection	28	5	0	1	25	2	34	4	3	1	28	2
Other non-CV	4	2	0	1	2	1	9	2	2	0	4	3

Values are n (%) or n.

Abbreviations as in Table 1.

a Investigator opinion taken from adjudication forms—this is different from adjudicated sudden death.

### Place of death

Of 377 deaths, 190 (50%) occurred in hospital >24 hours after hospitalization, 34 (9%) within 24 hours of hospitalization, 29 in hospice or residential care, 107 (28%) at home, and 17 in other places, such as a street, park, or shop (Table 4). The distribution of place of death was similar for patients assigned to FDI and to usual care.

### Cancer

The protocol excluded patients with cancer and a life expectancy of <2 years. Six patients had a record of cancer within 5 years before randomization. During the trial, 60 patients (12 women) had cancer hospitalizations or death, of which 9 occurred in the first year, 18 in the second; 15 in the third, and 18 thereafter. The most frequent cancers were gastrointestinal (n = 9 in each group), including gastroesophageal (n = 2), pancreas and bile duct (n = 5), small bowel (n = 1), and colorectal (n = 10), followed by lung (n = 11), bladder (n = 6), and prostate (n = 6). Values for TSAT, ferritin, and hemoglobin for those who did or did not develop cancer were similar except for cancer of the pancreas or bile duct, 3 of whom had a serum ferritin >100 μg/L. Similar numbers of patients randomized to usual care (n = 24) and to FDI (n = 36) had cancer events during follow-up.

## Discussion

For patients with HFrEF and iron deficiency enrolled in IRONMAN, a recurrent-events analysis found that FDI reduced unplanned hospitalizations with similar effects on CV and non-CV events. Patients assigned to FDI had slightly fewer CV deaths, but that did not attain statistical significance and the trial was not powered for that outcome.

Correcting iron deficiency might improve the function of many organs, reducing the incidence of a variety of problems that cause hospitalizations. For example, improved cardiac function might reduce the risk of atrial fibrillation, and improved immune function might reduce the risk of infection. Alternatively, correcting iron deficiency might increase a patient’s general resilience, so that even if a problem develops, they are less likely to need hospitalization (Central Illustration). For example, frailer patients who develop atrial fibrillation or an infection might not respond to simple measures and therefore require hospitalization, whereas those who are more resilient might not. The principal mechanism of benefit of iron therapy is likely to vary among patients, as well as over time and by context, for example, whether or not they were exposed to an infection.

Conventionally, CEACs report only the main cause of events.^5^ A different approach was adopted in IRONMAN, recognizing that patients with multiple medical conditions often have several problems that conspire to cause an event.^10^ For example, a chest infection may cause atrial fibrillation leading to worsening heart failure. Should the primary event be considered the initial trigger, the final consequence, or the best therapeutic target? Decisions may be biased by the specialties of the adjudicators, which may partly account for the high proportion of heart failure events reported by the IRONMAN CEAC. Reporting main and contributory causes of hospitalizations might make it easier to reach a consensus and avoids assumptions about what might be an effective intervention to prevent an event. For example, one-third of sudden deaths occurred in the context of worsening heart failure, which is not surprising, because increasing atrial and ventricular pressures and volumes leading to congestion are a major stimulus to arrhythmias.^18^ Treating congestion with mineralocorticoid receptor antagonists, sodium-glucose cotransporter 2 inhibitors, diuretics, and other guideline-recommended medicines might reduce the risk of sudden death and increase longevity, whereas implanting a defibrillator might be inappropriate for patients with worsening heart failure.^19^ Only 4% of patients died suddenly without worsening heart failure as a contributory factor during the course of the IRONMAN trial.

Worsening heart failure caused or contributed to 38% of hospitalizations and clearly remains an important therapeutic target for HFrEF despite improvements in therapy. Managing congestion is one of the most difficult tasks facing heart failure specialists, to ensure sufficient diuresis but avoid intravascular volume depletion and worsening renal function.^20^ AKI reflects increasing venous congestion, low arterial pressure, treatment with renin-angiotensin-aldosterone inhibitors, and intensive diuretic therapy. It usually occurs on a background of chronically impaired renal function but may also be exacerbated by infection. Fear of renal injury, leading to lower diuretic doses and inadequate diuresis, may prolong hospitalizations and increase mortality.^21^^,^^22^ Sodium-glucose cotransporter 2 inhibitors might improve control of congestion and reduce the incidence of AKI, leading to better diuretic management.^23^ Also, congested lungs are more prone to infection. Prompt treatment of infection might reduce the risk of hospitalization for heart failure. Many treatments that reduce hospitalizations for heart failure, perhaps now including correction of iron deficiency, also reduce hospitalizations for respiratory infections.^24^

Patients randomized to FDI were less likely to be hospitalized for heart failure. Correcting iron deficiency increases hemoglobin, which enhances systemic oxygen delivery for any given cardiac output. This could both improve myocardial oxygen delivery and reduce myocardial workload. Iron is an essential element for skeletal and cardiac myoglobin, which are important for oxygen uptake. Large amounts of adenosine triphosphate (ATP) are required for normal function of the heart, exercising skeletal muscle, kidney, and brain. Correcting iron deficiency may improve mitochondrial biogenesis and the efficiency of the electron transport chain, reducing free-radical production and improving synthetic capacity for ATP. For the heart, this may improve contractile function and reduce arrhythmias.^25^ For the kidney, this may improve water and salt excretion and reduce the risk of AKI.^26^

Patients randomized to FDI tended to have more hospitalizations associated with bleeding. Iron deficiency is associated with increased platelet production, which might be reversed by iron supplements, leading to an increased risk of bleeding but, potentially, a reduction of acute coronary syndrome events.^27^ However, for those assigned to FDI, there was a small increase in hospitalizations for stroke, which was not further adjudicated as hemorrhagic or ischemic. A randomized trial comparing different doses of iron sucrose for patients on hemodialysis found no difference in fatal or nonfatal strokes.^28^ Mendelian randomization studies have reported conflicting evidence on stroke risk for people genetically predisposed to having higher body iron.^29^^,^^30^ However, cohort studies suggest a doubling in the risk of stroke for those aged ≥65 years with iron deficiency.^31^ Data on strokes from other randomized trials of intravenous iron should be reported.

Infection contributed to even more hospitalizations than did worsening heart failure. Most infections were respiratory, urinary, or due to cellulitis. Patients assigned to FDI had fewer hospitalizations for respiratory infection. This could reflect fewer infections. Alternatively, fewer hospitalizations may reflect greater resilience to infection, resulting in a less severe clinical course, better response to antibiotics, or a lower risk of complications, such as atrial fibrillation or AKI, allowing the patient to recover without being hospitalized. In 2 small randomized trials of patients with chronic lung disease, intravenous iron improved symptoms and exercise capacity,^32^^,^^33^ although this might not translate into a reduction in hospitalizations. Correcting iron deficiency might improve immune responses to vaccination as well as to infection.^34^^,^^35^ Prompt management of infection for patients with heart failure might reduce hospitalizations for both infection and heart failure.

No difference in all-cause mortality was observed for patients assigned to FDI or usual care. A larger trial unconfounded by the Covid-19 response and focusing on patients with TSAT <20% would be required to confirm or refute the trends in CV and all-cause mortality observed in IRONMAN.^2^ About 70% of deaths were CV and were predominantly due to worsening heart failure. More than one-half of all deaths occurred in hospital. Although investigators reported that about one-half of CV deaths were unexpected, only one-half of unexpected deaths were classified as sudden by the CEAC, with many others classified as due to heart failure. This suggests that a fairly low proportion of deaths were likely to be prevented by implantable defibrillators and further highlights the complexity of ascribing single causes to hospitalizations and deaths.

About 5% of patients had a cancer-related hospitalization or death, which, at 1.5% per year, is half that expected in the general population of a similar age.^36^ Some cancers would not have required hospitalization and others will have been missed, a problem exacerbated by the Covid-19 measures. Many cancer-related events occurred in the first 2 years of the trial, suggesting that some undiagnosed cancers might have been present before randomization. Screening for gastrointestinal cancers is done routinely for the general population aged ≥60 years in many countries.^37^ Testing of urine and feces for blood would be a simple addition to the routine work-up for patients with heart failure who are about to receive intravenous iron. For those who require frequent doses of iron, further investigation should be considered.

### Study Limitations

Planned research visits were prohibited for long periods due to the Covid-19 response, restricting administration of further doses of FDI. Few patients enrolled in IRONMAN were hospitalized or died from Covid-19, possibly because of effective shielding of this vulnerable population. However, the rate of heart failure hospitalization in the United Kingdom and elsewhere declined during the Covid-19 period, either because patients were reluctant to be hospitalized or because health care staff made extra efforts to manage patients without hospitalization,^38^ which may have affected our results. For many clinical trials, investigators, company representatives or administrative staff filter nonfatal events and present only those that might constitute an event to the CEAC. In IRONMAN, the CEAC reviewed all hospitalizations that constituted a serious adverse event. To make this feasible with limited resources, after an initial training period to ensure consistency among CEAC members, most endpoints were adjudicated by 1 reviewer, who referred events to the entire committee only when uncertain about adjudication. If all events had been reviewed by 2 or more reviewers, the results might have been slightly different. The endpoint categories were decided before starting the adjudication process. With greater experience, modification of the classification system to include gastrointestinal events or to classify the nature of infections better should be considered. The trial was not powered for secondary or subgroup analyses, and some statistical differences may have occurred by chance owing to multiple testing. Our results should be confirmed or refuted in other data sets, but they are consistent with the effects of intravenous iron on CV hospitalizations observed in other randomized trials.^1^

## Conclusions

For patients with HFrEF randomized in IRONMAN, administration of FDI reduced the rate of unplanned hospitalization for both CV (mainly heart failure) and non-CV (mainly respiratory disease and infection) causes. Correcting iron deficiency might increase resistance or resilience to a broad range of problems that might otherwise cause hospitalizations. Better control of congestion and prompt treatment of infection might have the greatest impact on reducing hospitalization for patients similar to those enrolled in IRONMAN. However, whether correcting iron deficiency has an effect on mortality, or on its mode or cause, remains uncertain.

## Funding Support and Author Disclosures

The IRONMAN trial was funded by the British Heart Foundation (grant CS/15/1/31175). Pharmacosmos supplied FDI and gave additional financial support. Dr Cleland has received funding paid to University of Glasgow for other clinical trials and registries from Bristol Myers Squibb and CSL-Vifor; has received consulting fees paid to University of Glasgow from Pharmacosmos, CSL-Vifor, and Biopeutics; has received honoraria for lectures and support for attending meetings paid to University of Glasgow from Pharmacosmos; is chairperson for data monitoring committees for ADAPT-CRT, CMR-Guide, and PROTECT-HF; and has shares or stock options in HeartFelt (noninvasive monitoring) and Viscardia (synchronous diaphragmatic pacing). Dr Pellicori has received consulting fees from Pharmacosmos, Vifor, and Caption Health; has received honoraria for lectures from AstraZeneca; and has received support for attending meetings from Pharmacosmos. Dr Graham has received personal consulting fees from Vifor; and has received personal support for attending meetings from Pharmacosmos. Dr Petrie has received grants/contracts from Boehringer Ingelheim, Roche, SQ Innovations, AstraZeneca, Novartis, Novo Nordisk, Medtronic, Boston Scientific, and Pharmacosmos; has received consulting fees from Akero, Applied Therapeutics, Amgen, AnaCardio, Biosensors, Boehringer Ingelheim, Novartis, AstraZeneca, Novo Nordisk, Abbvie, Bayer, Horizon Therapeutics, Takeda, Cardiorentis, Pharmacosmos, Siemens, Eli Lilly, Vifor, New Amsterdam, Moderna, Teikoku, LIB Therapeutics, and 3R Lifesciences; and has participated on data safety monitoring boards for Moderna and Teikoku. Dr Ahmed has received honoraria for lectures from Pharmacosmos. Dr Squire has received grants/contracts from the British Heart Foundation. Dr Ludman has received honoraria for lectures from AstraZeneca; and is chairperson for the British Cardiovascular Society Guidelines and Practice Committee. Dr Japp has received personal consulting fees from Pharmacosmos; has received honoraria for educational events from Novartis and AstraZeneca; has received support for attending meetings from Novartis; has participated on an advisory board for Pharmacosmos; and is Clinical Lead and Chair for Heart Failure Hub Scotland. Dr Al-Mohammad has received personal honoraria for lectures from AstraZeneca, Janssen, Takeda, and Pharmacosmos; has participated on advisory boards for Novartis, Pharmacosmos, AstraZeneca, Boehringer Ingelheim, and Lilly; is a safety monitoring board member for the REACH-HFpEF Study and a UK-HFpEF registry Executive Steering Committee member; and has received equipment from AstraZeneca. Dr Clark is Chair of the Programme Committee for BCS. Dr Szwejkowski has received support for attending meetings from AstraZeneca. Dr McMurray has received personal consulting fees from Alynylam Pharmaceuticals, Bayer, BMS, Ionis Pharmaceuticals, Novartis, Regeneron Pharmaceuticals, and River 2 Renal Corp; is a director of Global Clinical Trial Partners; has received personal honoraria for lectures from Abbott, Alkem Metabolics, AstraZeneca, Blue Ocean Scientific Solutions Ltd., Boehringer Ingelheim, Canadian Medical and Surgical Knowledge, Emcure Pharmaceuticals, Eris Lifesciences, European Academy of CME, Hikma Pharmaceuticals, Imagica Health, Intas Pharmaceuticals, J.B. Chemicals & Pharmaceuticals, Lupin Pharmaceuticals, Medscape/Heart.Org, ProAdWise Communications, Radcliffe Cardiology, Sun Pharmaceuticals, The Corpus, Translation Research Group, and Translational Medicine Academy; has participated on a data safety monitoring board for George Clinical PTY; and has received funding paid to University of Glasgow for involvement with the following companies/trials: AstraZeneca (DAPA-HF, DELIVER, DETERMINE, DAPA-Resist, DAPA-CKD), Amgen (ATOMIC-HF, COSMIC-HF, GALACTIC-HF), Bayer (FINEARTS), Cardurion (company advisory board), Cytokinetics (GALACTIC-HF), GlaxoSmithKline (ASCEND-D, ASCEND-ND), KBP Biosciences (scientific advisor), and Novartis (PARAGON-HF, PARADISE-MI, PERSPECTIVE, PARACHUTE-HF). Drs Thomson and Robertson have received grants paid to University of Glasgow from the British Heart Foundation and Pharmacosmos. Dr Ford has received grants paid to University of Glasgow from the British Heart Foundation and Pharmacosmos and study drug from Pharmacosmos. Dr Philip Kalra has received funding from Pharmacosmos; has received grants from CSL Vifor, Astellas, Evotec, Pharmacosmos, and Unicyte; has received consulting fees from AstraZeneca, CSL Vifor, Unicyte, and UCB; has received honoraria for lectures from CSL Vifor, AstraZeneca, Pfizer, Pharmacosmos, Napp, and Bayer; and has received support for attending meetings from Pharmacosmos and CSL Vifor. Dr Paul Kalra has received grants paid to University of Glasgow from the British Heart Foundation and Pharmacosmos and to Portsmouth Hospitals University NHS Trust from Pharmacosmos; has received personal consulting fees from Amgen, Boehringer Ingelheim, Pharmacosmos, Servier, and CSL Vifor; has received personal honoraria for lectures from AstraZeneca, Bayer, Novartis, Pfizer, Pharmacosmos, CSL Vifor, and Amgen; has received support for attending meetings from Pharmacosmos; has participated on data safety monitoring boards for the STOP-ACE and EMPRESS-MI trials; and has been Chair-Elect, Chair, and Past Chair for the British Society for Heart Failure. All other authors have reported that they have no relationships relevant to the contents of this paper to disclose.
