# Supplementary material for: Adjudication of Hospitalizations and Deaths in the IRONMAN Trial of Intravenous Iron for Heart Failure
Source: J Am Coll Cardiol. 2024 Oct 29;84(18):1704–17. doi: 10.1016/j.jacc.2024.08.052 (PMC11496827; doi:10.1016/j.jacc.2024.08.052)
Supplement: Supplemental Material [file mmc1.docx]

**Adjudication of Hospitalizations and Deaths in the IRONMAN Trial of Intravenous Iron for Heart Failure**

| **Page 1** | Supplemental Table 1 | Clinical endpoints classification system. |
| --- | --- | --- |
| **Page 2** | List of Committees |  |
| **Page 3** | List of Sites & Investigators |  |

**Supplemental Table 1**

Clinical endpoints classification system. Each event was attributed to a single main cause, but multiple contributory causes could be assigned. For instance, a patient might be admitted with worsening heart failure but with a respiratory infection that had triggered atrial fibrillation and acute kidney injury. Conventionally, the admission might have been attributed solely to heart failure, which would provide an incomplete and potentially misleading perspective of the reasons for admission.

| **Death** | **Hospitalisation** |
| --- | --- |
| **Cardiovascular** | **Cardiovascular** |
| Acute Coronary Syndrome | Acute Coronary Syndrome |
| Sudden Cardiac | Arrhythmias   1. Ventricular 2. Supraventricular |
| End-Stage Heart Failure | Worsening Heart Failure   1. Main reason 2. Contributory to CV admission 3. Contributory to non-CV admission 4. Late complication |
| Cardio-renal / Acute Kidney Injury | Cardio-renal / Acute kidney injury |
| Stroke | Stroke |
| Haemorrhage | Haemorrhage |
| Due to Cardiovascular Medicines & Procedures | Due to Cardiovascular Medicines & Procedures |
| Pulmonary Embolism | Pulmonary Embolism |
| Other | Other |
| **Non-Cardiovascular** | **Non-Cardiovascular** |
| Cancer | Cancer |
| Respiratory | Respiratory |
| Infection | Infection |
| End-Stage Renal Disease | End-Stage Renal Disease |
| Trauma and Non-CV Procedures | Trauma and Non-CV Procedures |
| Infection | Infection |
| Other | Other |
| **Undetermined** | **Undetermined** |

CV = cardiovascular

**LIST OF COMMITTEES**

CLINICAL ENDPOINTS COMMITTEE

Professor John GF Cleland, Dr Fraser Graham, Dr Pierpaolo Pellicori

Administrative support: Dr Eleanor Dinnett, Amanda Reid, Claire Brunton, Elizabeth Thomson

CLINICAL TRIALS UNIT AND DATA AND STATISTICAL CENTRE

Robertson Centre for Biostatistics, University of Glasgow: Elizabeth Thomson (Project Management), Professor Ian Ford (Study Director), Michele Robertson, Nicola Greenlaw and Kirsty Wetherall (Biostatistics), Ross Clarke, Christopher Graham, Sharon Kean, Alan Stevenson and Robbie Wilson (eCRF), Sarah Boyle and John McHugh (Data Management), Lisa Hall and Joanne Woollard (Projects Administration), Claire Brunton, Dr Eleanor Dinnett and Amanda Reid (Endpoints Coordination)

NURSING AND ADMINISTRATIVE SUPPORT

Serena Howe, Portsmouth Hospitals University NHS Trust, Portsmouth, UK

Jill Nicholls, NHS Tayside, Dundee, UK

Anna Cunnington, Portsmouth Hospitals University NHS Trust, Portsmouth, UK

SPONSOR SUPPORT

From NHS Greater Glasgow and Clyde, Glasgow, UK: Dr Elizabeth Douglas, Dr Margaret Fegen, Dr Marc Jones, Dr Sheila McGowan, Dr Barbara Ross, Dr Pamela Sandu, Pamela Surtees

From University of Glasgow, Glasgow, UK: Dr Debra Stuart

**SITES & INVESTIGATORS**

Paul Kalra, Elena Cowan, Charlotte Turner, Rosalynn Austin, Queen Alexandra Hospital, Portsmouth, UK

Rebeca Lane, Paula Rogers, Royal Brompton and Harefield Hospital, London, UK

Paul Foley, Badri Chandrasekaran, Eva Fraile, Lynsey Kyeremeh, Great Western Hospital, Swindon, UK

Fozia Ahmed, Manchester Royal Infirmary, Manchester, UK

Mark Petrie, Lorraine McGregor, Joanna Osmanska, Fraser Graham, Glasgow Royal Infirmary, Glasgow, UK

Ninian Lang, Barbara Meyer, Faheem Ahmad, Joanna Osmanska, Queen Elizabeth University Hospital, Glasgow, UK

Iain Squire, Jude Fisher, Glenfield Hospital, Leicester, UK

Philip Kalra, Christina Summersgill, Katarzyna Adeniji, Rajkumar Chinnadurai, Salford Royal Hospital, Salford, UK

Andrew Ludman, Lisa Massimo, Clare Hardman, Daisy Sykes, Royal Devon and Exeter Hospital, Exeter, UK

Peter Cowburn, Sarah Frank, Simon Smith, University Hospital Southampton, Southampton, UK

Alan Japp, Mohamed Anwar, Beth Whittington, Royal Infirmary of Edinburgh, Edinburgh, UK

Alison Seed, Blackpool Victoria Hospital, Blackpool, UK

Robin Ray, Vennessa Sookhoo, Sinead Lyons, St. George’s Hospital, London, UK

Abdallah Al-Mohammad, Janet Middle, Kay Housley, Northern General Hospital, Sheffield, UK

Andrew Clark, Jeanne Bulemfu, Castle Hill Hospital, Hull, UK

Christopher Critoph, Royal Bournemouth Hospital, Bournemouth, UK

Victor Chong, Stephen Wood, University Hospital Crosshouse, Kilmarnock, UK

Benjamin Szwejkowski, Chim Lang, Jackie Duff, Susan MacDonald, Ninewells Hospital, Dundee, UK

Rebekah Schiff, Guy’s and St Thomas’ Hospital, London, UK

Patrick Donnelly, Ulster Hospital, Dundonald, UK

Thuraia Nageh, Swapna Kunhunny, Southend University Hospital, Southend, UK

Mark Petrie, Roy Gardner, Marion McAdam, Elizabeth McPherson, Golden Jubilee National Hospital, Clydebank, UK

Prithwish Banerjee, Eleanor Sear, Nigel Edwards, University Hospital Coventry, Coventry, UK

Jason Glover, Basingstoke and North Hampshire Hospital, Basingstoke, UK

Pierpaolo Pellicori, Clare Murphy, Royal Alexandra Hospital, Paisley, UK

Justin Cooke, Chesterfield Royal Hospital, Chesterfield, UK

Charles Spencer, New Cross Hospital, Wolverhampton, UK

Mark Francis, Victoria Hospital, Kirkcaldy, UK

Iain Matthews, Hayley McKie, Wansbeck General Hospital, UK

Andrew Marshall, Janet Large, Jenny Stratford, District General Hospital, Eastbourne, UK

Piers Clifford, Sara Tavares, Hammersmith Hospital, London, UK

Christopher Boos, Poole Hospital, Poole, UK

Philip Keeling, Debbie Hughes, Torbay Hospital, Torquay, UK

Aaron Wong, Deborah Jones, Alex James, Rhys Williams, Princess of Wales Hospital, Bridgend, UK

Stephen Leslie, Jim Finlayson, Raigmore Hospital, Inverness, UK

Piers Clifford, Wycombe Hospital, High Wycombe, UK

Andrew Hannah, Aberdeen Royal Infirmary, Aberdeen, UK

Philip Campbell, Royal Gwent Hospital, Newport, UK

John Walsh, Jane Quinn, Nottingham University Hospital, Nottingham, UK

Callum Chapman, West Middlesex University Hospital, Isleworth, UK

Susan Piper, Sheetal Patale, King’s College Hospital, London, UK

Preeti Gupta, Victor Sim, Lucy Knibbs, University Hospital Llandough, Penarth, UK

Kristopher Lyons, Antrim Area Hospital, Antrim, UK

Lana Dixon, Royal Victoria Hospital, Belfast, UK

Colin Petrie, University Hospital Monklands, Airdrie, UK

Yuk-ki Wong, St Richard’s Hospital, Chichester, UK

Catherine Labinjoh, Forth Valley Royal Hospital, Larbert, UK

Simon Duckett, Ian Massey, Royal Stoke University Hospital, Stoke-On-Trent, UK

Henry Savage, Sofia Matias, Jonaifah Ramirez, Basildon University Hospital, Basildon, UK

Charlotte Manisty, Ifza Hussain, St Bartholomew’s Hospital, London, UK

Rajiv Sankaranarayanan, Gershan Davis, Aintree University Hospital, Liverpool, UK

Samuel McClure, John Baxter, Sunderland Royal Hospital, Sunderland, UK

Eleanor Wicks, John Radcliffe Hospital, Oxford, UK

Jolanta Sobolewska, Royal Oldham Hospital, Oldham, UK

Jerry Murphy, Darlington Memorial Hospital, Darlington, UK

Ahmed Elzayat, Alastair Cooke, Doncaster Royal Infirmary, Doncaster, UK

Jay Wright, Liverpool Heart and Chest Hospital, Liverpool, UK

Simon Williams, Wythenshawe Hospital, Manchester, UK

Amal Muthumala, North Middlesex University Hospital, London, UK

Parminder Chaggar, Sue Webber, Royal Cornwall Hospital, Truro, UK

Gethin Ellis, Mandie Welch, Royal Glamorgan Hospital, Llantrisant, UK

Sudantha Bulugahapitiya, Bradford Royal Infirmary, Bradford, UK

Thomas Jackson, Salisbury District Hospital, Salisbury, UK

Tapesh Pakrashi, Kingston Hospital, Kingston Upon Thames, UK

Ameet Bakhai, Vinodh Krishnamurthy, Barnet Hospital, Barnet, UK

Reto Gamma, Broomfield Hospital, Chelmsford, UK

Susan Ellery, Royal Sussex County Hospital, Brighton, UK

Charlotte Manisty, University College London Hospital, London, UK

Geraint Jenkins, Gladdys Thomas, Morriston Hospital, Swansea, UK

Angus Nightingale, Bristol Royal Infirmary, Bristol, UK
